# Association of myopia and parapapillary choroidal microvascular density in primary open-angle glaucoma

**DOI:** 10.1371/journal.pone.0317881

**Published:** 2025-02-25

**Authors:** Yanin Suwan, Sunee Chansangpetch, Masoud Aghsaei Fard, Pakinee Pooprasert, Kamolwat Chalardsakul, Thanatporn Threetong, Siripa Tipparut, Thiraporn Saaensupho, Apichat Tantraworasin, Sahar Hojati, Raheleh Kafieh, Hajar Danesh, Purit Petpiroon, Wasu Supakontanasan

**Affiliations:** 1 Department of Ophthalmology, Ramathibodi Hospital, Mahidol University, Bangkok, Thailand; 2 Department of Ophthalmology, Faculty of Medicine, Chulalongkorn University and King Chulalongkorn Memorial Hospital, Thai Red Cross Society, Bangkok, Thailand; 3 Center of Excellence in Glaucoma, Chulalongkorn University, Bangkok, Thailand; 4 Farabi Eye Hospital, Tehran University of Medical Sciences, Tehran, Iran; 5 Department of Ophthalmology, Burapha University Hospital, Chonburi, Thailand; 6 Surgery and Clinical Epidemiology and Clinical Statistic Center, Faculty of Medicine, Chiang Mai University, Chiang Mai, Thailand; 7 Clinical Surgical Research Center, Chiang Mai University, Chiang Mai, Thailand; 8 Medical Image and Signal Processing Research Center, School of Advanced Technologies in Medicine, University of Medical Sciences, Isfahan, Iran; 9 Engineering Department at Durham University, Durham, United Kingdom; 10 Department of Electrical and Biomedical Engineering, Faculty of Engineering and Technology, Shahid Ashrafi Esfahani University, Isfahan, Iran; Alexandria University Faculty of Medicine, EGYPT

## Abstract

**Background/Aims:**

To compare parapapillary choroidal microvascular (PPCMv) densities between myopic eyes with and without glaucoma.

**Methods:**

In this retrospective study, OCTA images (4.5 × 4.5 mm) were obtained using a commercial spectral-domain OCTA system. PPCMv density was calculated in inner and outer annuli using customized software. Marginal model of generalized estimating equations was established to adjust for confounding factors and intraclass correlations.

**Results:**

This study included 35 myopic eyes with glaucoma (MG), 96 non-myopic eyes with glaucoma (NMG) matched for visual field mean deviation, 37 myopic eyes without glaucoma (MNG), and 73 control eyes from three tertiary centers. The participant ages were (mean [standard deviation, SD]) 57.43 [11.49], 60.40 [10.07], 52.84 [9.35], and 54.74 [12.07] years. Inner and outer annular PPCMv densities (mean [SD]) decreased in the following order: control (0.15 [0.04] and 0.12 [0.04]), MNG (0.14 [0.08] and 0.12 [0.08]), NMG (0.09 [0.05] and 0.07 [0.04]), and MG (0.09 [0.04] and 0.07 [0.03]). The mean differences in PPCMv density between glaucoma groups (NMG and MG) and the control group (mean difference [95% confidence interval]) were −0.06 (−0.08 to −0.04, P < 0.001 for inner whole annular PPCMv density in NMG vs control) and −0.07 (−0.10 to −0.04, P < 0.001 for inner whole annular PPCMv density in MG vs control), consistent across all regions of interest (ROIs). No significant interaction was observed between glaucoma and myopia after adjustment for potential confounders (P > 0.112).

**Conclusions:**

Parapapillary choroidal microvascular density attenuation tends to be greater in eyes with glaucoma than in eyes with myopia.

## Introduction

Myopia is the most common refractive error, affecting more than 1.4 billion people worldwide, and its prevalence is expected to increase to 4.7 billion by 2050 [1]. Myopia is associated with increased risks of ophthalmic complications such as glaucoma. For example, it reportedly causes a two- to threefold increase in the risk of open-angle glaucoma (OAG) [2,3]. The relationships of myopia with various aspects of glaucoma pathogenesis have been elucidated in many clinical studies [3,4]. Other than intraocular pressure (IOP)-induced mechanical damage, there remains a lack of clarity regarding the roles of optic disc-associated microvascular alterations in the development of myopia-related OAG. This lack of clarity leads to challenges in diagnosing, monitoring, and managing glaucoma among patients with myopia.

Previous studies using various techniques demonstrated reduced retinal blood flow in patients with high myopia [5,6]. Myopic eyes show decreases in superficial and deep macular and radial parapapillary capillary vessel densities, along with increased densities inside the optic disc and elongated foveal avascular zones [7–12]. These changes may serve as indicators of future progression to pathologic myopia. The loss of photoreceptor cells, which are nourished by the choroid, is a major cause of visual impairment in pathologic myopia. Therefore, any choroidal dysfunction has a detrimental effect on myopic eyes. In degenerative myopia, both choroidal and retinal blood flow appear to be reduced; these changes may be partly related to increased vascular resistance or adaptive responses to axial elongation [13–15].

Although there has been extensive research concerning superficial microvascular alterations in various types of glaucoma and myopia [16,17], few have explored the choroidal microvasculature. This lack of research emphasis can be attributed to the limited penetration characteristics of the shorter wavelengths used in spectral-domain OCTA. Additionally, the slow blood flow in the choriocapillaris may not generate a detectable decorrelation signal, and previous studies have mainly focused on qualitative assessments [18–20]. However, the use of OCTA to evaluate glaucoma is not affected by the low reflectance of the retinal nerve fiber layer (RNFL) or structural deformation of the optic disc. Therefore, the parapapillary choroidal microvasculature is increasingly important as a potential marker for the perfusion of deep optic nerve head (ONH) structures in glaucoma [18,19]. There is speculation that reduced juxtapapillary choroidal volume contributes to vascular insufficiency in the ONH, resulting in glaucoma [21]. In this study, we used OCTA to evaluate choroidal vessel density in myopic eyes with and without OAG.

## Patients and methods

This retrospective study was conducted in Ramathibodi, King Chulalongkorn Memorial Hospitals. The study adhered to the Health Insurance Portability and Accountability Act and was performed in accordance with the tenets of the Declaration of Helsinki. The study was approved by the Institutional Review Board (IRB) of the Faculty of Medicine Ramathibodi Hospital, Mahidol University, Bangkok, Thailand (IRB number: COA. MURA2022/44) and the Faculty of Medicine Chulalongkorn University, Bangkok, Thailand (IRB number: COA No. 0299/2023) which waived the requirement for written informed consent from participants due to the retrospective nature of the study. 

### Participants

Study participants, including glaucoma patients with or without myopia, myopia patients without glaucoma, and healthy controls diagnosed from January 2021 to Dcember 2021, were enrolled. The inclusion criteria for all groups were best-corrected visual acuity ≥ 20/40 and age > 18 years. Myopia was regarded as a spherical equivalent refractive error of -3 diopters or worse.

OAG was diagnosed based on a glaucomatous appearance of the optic nerve (neuroretinal rim thinning or notching, and retinal nerve fiber layer [RNFL] defects) as determined by glaucoma specialists on dilated fundus examination, the presence of an open anterior chamber angle, RNFL thinning on optical coherence tomography (OCT), and visual field defects that were correlated with ONH findings. Glaucomatous visual field changes included the presence of ≥ 3 abnormal points with a probability of being normal of P-value < 5%, with at least one of these points depressed to the level of < 1%; a glaucoma hemifield test result outside the normal limits; or a pattern standard deviation of P-value < 5%. OAG patients were classified according to stage as follows: mild, mean deviation (MD)>−6 dB; moderate, − 6 to − 12 dB, and severe, ≤−12 dB.

Healthy controls exhibited an IOP of ≤ 21 mmHg without a history of increased IOP, an open anterior chamber angle, the absence of glaucomatous disc characteristics, OCT-measured global circumpapillary RNFL thickness within the 95% confidence interval (CI) of the mean, and the absence of visual field defects as described above.

Exclusion criteria for all eyes included the presence of any media opacity that prevented high-quality OCTA scans, a history of ocular surgery other than uncomplicated cataract surgery, chronic use of ocular or systemic steroids, diabetes mellitus, non-glaucomatous optic neuropathy, and cardiovascular diseases other than uncomplicated systemic hypertension.

### Clinical examinations

We recorded demographic data and clinical characteristics, including best-corrected visual acuity, IOP (measured by Goldmann applanation tonometry), axial length (assessed with IOL Master [Carl Zeiss Meditec, Jena, Germany]), and systolic and diastolic blood pressures. Automated perimetry was performed on the Humphrey Visual Field Analyzer (Humphrey Instruments Model 740; Carl Zeiss Meditec, Dublin, CA, USA) using the 24-2 Swedish Interactive Thresholding Algorithm standard program.

### OCT and OCTA image acquisition and scanning protocol

OCT and OCTA images were acquired using the AngioVue imaging system (AngioVue Software V.2011.1.1.151; Optovue, Inc., Fremont, CA, USA). Standard circumpapillary RNFL thickness was measured, and RNFL thickness values (global and sector-specific) were recorded.

**Fig 1:**
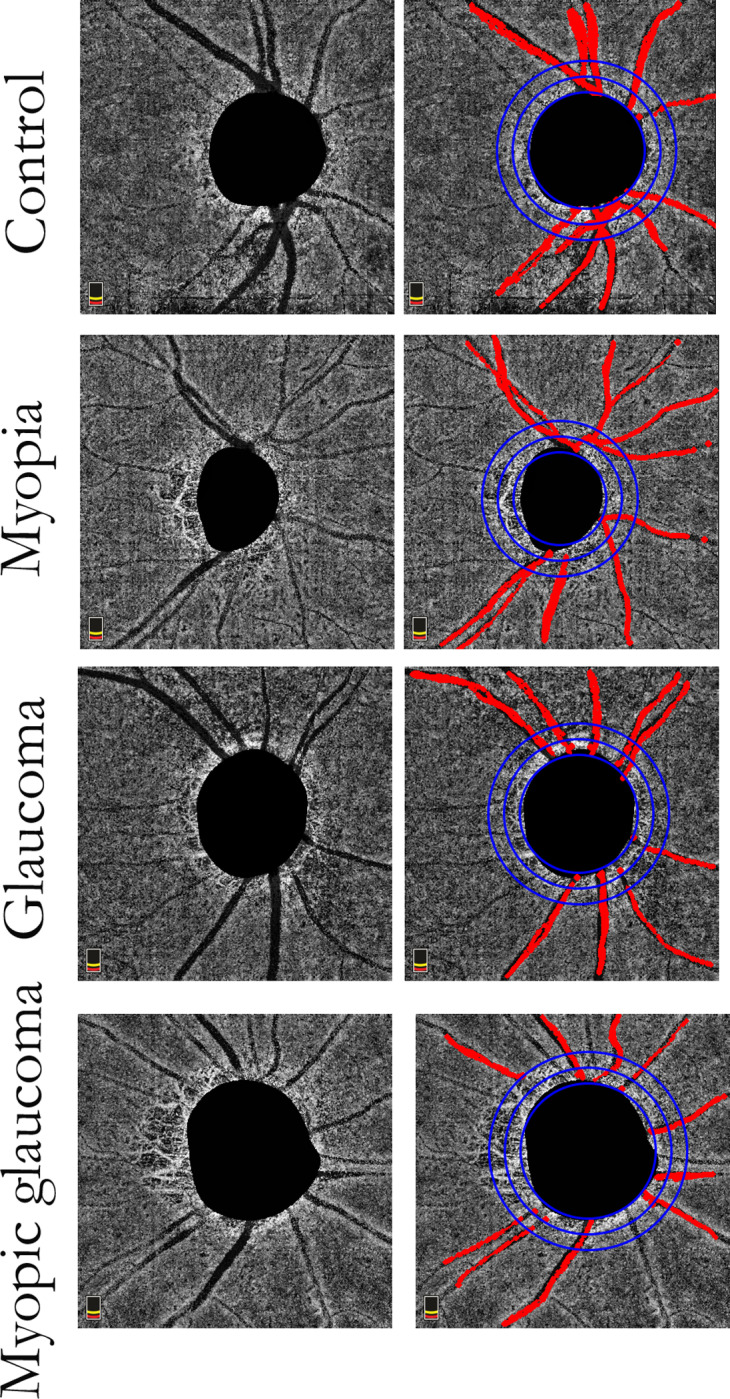
Parapapillary choroidal microvasculature in eyes with myopia and glaucoma (inner/outer whole annulus PPCMv densities=  0.092/0.069), glaucoma without myopia (0.092/0.071), and myopia without glaucoma (inner/outer whole annulus PPCMv densities =  0.139/0.119), as well as control (inner/outer whole annulus PPCMv densities =  0.148/0.122) eyes. Left: OCTA images of deep parapapillary vessels after removal of large vessels; (right) OCTA images of deep parapapillary microvasculature after removal of retinal vessel shadows (red), overlaid by three concentric circles (each exhibiting a width of 0.5 mm), in four study eyes. OCTA, optical coherence tomography angiography.

OCTA images were obtained with a commercial spectral-domain OCT system (RTVue XR, version 2018.0.0.18; Optovue, Inc.) using a 4.5- × 4.5-mm scan centered on the optic disc. Parapapillary choroidal microvascular (PPCMv) densities were analyzed using customized MATLAB software (The MathWorks, Inc., Natick, MA, USA) after removal of the densities of large retinal vessels and exclusion of data from inside the optic disc, as previously reported.^18,19^ Three concentric circles were superimposed on the en face image (Fig 1).

The inner circle was automatically drawn around the disc location; this location was highlighted in black by the Optovue OCTA and its diameter varied across images. Subsequently, the middle and outer circles were positioned concentrically with respective diameters 1 mm and 2 mm larger than the inner circle. Data from the inner and outer coaxial annular regions of interest (ROIs; each exhibiting a width of 0.5 mm), along with their upper and lower halves, were utilized to calculate PPCMv density values in the study groups.

Images with significant background noise, motion artifacts, a signal strength index of < 40, or segmentation errors within the ROIs were excluded.

### Statistical analysis

After the distributions of numerical data had been assessed using the Shapiro–Wilk test, normally distributed variables were described as means and standard deviations (SDs), whereas non-normally distributed variables were described as medians and interquartile ranges. Categorical variables were compared using the X^2^ test. Linear mixed models were used to evaluate groupwise differences in variables after adjustments for age, sex, axial length, visual field mean deviation (VF MD), and intraclass correlations. Both eyes of each participant were included in the analysis. All statistical analyses were performed with Stata software, version 14 (StataCorp, College Station, TX, USA). The a level (threshold for type 1 error) was regarded as.05 for all comparisons.

## Results

### Study population

In total, 248 eyes met the inclusion criteria. Seven eyes were excluded due to poor image quality. The distribution of eyes among groups was as follows: 35 eyes in the MG group (mean [SD] age, 57.43 [11.49] years), 96 eyes in the NMG group (mean [SD] age, 60.40 [10.07] years), 37 eyes in the MNG group (mean [SD] age, 52.84 [9.35] years), and 73 eyes in the control group (mean [SD] age, 54.74 [12.07] years). The sex distribution varied across the groups; the percentages of female participants were 60%, 48%, 22%, and 26% in the MG, NMG, MNG, and control groups, respectively (Table 1).

**Table 1:** Participant Clinical and Demographic Characteristics.

Total	Control	Myopia Without Glaucoma	Glaucoma Without Myopia	Myopia and Glaucoma	P Value
	73	37	96	35	
Age, years, mean (SD)	54.74 (12.07)	52.84 (9.35)	60.40 (10.07)	57.43 (11.49)	0.001
Sex, N (%)					
Male	19 (26.03)	8 (21.62)	46 (47.92)	21 (60.00)	
Female	54 (73.97)	29 (78.38)	50 (52.08)	14 (40.00)	0.000
AXL, mm, mean (SD)	23.38 (1.17)	26.27 (1.52)	23.83 (1.35)	26.29 (1.28)	0.000
SBP, mmHg, mean (SD)	125.82 (18.41)	124.00 (16.84)	134.93 (18.04)	125.23 (18.93)	0.001
DBP, mmHg, mean (SD)	75.49 (9.09)	76.73 (8.07)	77.16 (8.22)	75.14 (6.64)	0.478
SE, D, median (IQR)	0 (−0.12, 0)	−7 (−8.25, −5.5)	0 (0, 0.75)	−5.75 (−7.25, −4.5)	0.000
VF parameter					
MD, dB, median (IQR)	−0.61 (−1.76, 0)	−0.9 (−2.84, 0.05)	−4.08 (−9.26, −2.2)	−5.5 (−9.46, −2.23)	0.000
PSD, dB, median (IQR)	1.65 (1.43, 1.97)	1.68 (1.47, 1.99)	4.83 (2.17, 9.44)	6.62 (2.15, 12.19)	0.000
VFI, median (IQR)	99 (98, 99)	98 (97, 99)	90.5 (80.5, 98)	91 (76, 97)	0.000
Glaucoma severity, N (%)					0.000
Mild	–	–	63 (66.32%)	20 (57.14%)	
Moderate	–	–	14 (14.74%)	12 (34.29%)	
Severe	–	–	18 (18.95%)	3 (8.57%)	
OCT, µm, mean (SD)					
Global	103.51 (17.16)	103.69 (10.73)	78.86 (16.04)	80.43 (19.79)	0.000
Superior	128.18 (20.72)	127.00 (21.28)	95.41 (23.81)	95.86 (29.18)	0.000
Nasal	81.67 (19.63)	90.82 (23.13)	67.37 (16.82)	75.84 (19.29)	0.000
Inferior	136.06 (25.59)	124.88 (23.19)	88.01 (24.12)	87.41 (31.65)	0.000
Temporal	70.35 (12.80)	83.19 (48.55)	63.74 (15.56)	63.73 (14.01)	0.000
PPCMv densities, mean (SD)					
Inner whole annulus	0.148 (0.044)	0.139 (0.022)	0.092 (0.045)	0.092 (0.040)	0.000
Outer whole annulus	0.122 (0.036)	0.119 (0.078)	0.071 (0.036)	0.069 (0.032)	0.000
Inner hemi-superior	0.159 (0.054)	0.159 (0.093)	0.089 (0.050)	0.087 (0.053)	0.000
Outer hemi-superior	0.131 (0.044)	0.134 (0.086)	0.072 (0.045)	0.071 (0.041)	0.000
Inner hemi-inferior	0.138 (0.051)	0.119 (0.081)	0.095 (0.056)	0.096 (0.059)	0.000
Outer hemi-inferior	0.113 (0.039)	0.104 (0.077)	0.069 (0.039)	0.066 (0.038)	0.000

AXL, axial length; SD, standard deviation; VF, visual field; MD, mean deviation; IQR, interquartile range; PSD, pattern standard deviation; VFI, visual field index; SBP, systolic blood pressure; DBP, diastolic blood pressure; SE, spherical equivalent; OCT, optical coherence tomography; PPCMv, parapapillary choroidal microvascular.

There were significant differences among the four groups in terms of systolic blood pressure, spherical equivalent refractive error, axial length, global and sectoral RNFL thicknesses, and visual field indices. Moreover, glaucoma severity varied across the groups. Among participants in the NMG group, 63 (66.32%) had mild glaucoma, 14 (14.74%) had moderate glaucoma, and 18 (18.95%) had severe glaucoma. In contrast, among participants in the MG group, 20 (57.14%) had mild glaucoma, 12 (34.29%) had moderate glaucoma, and three (8.57%) had severe glaucoma. There were no significant differences in global RNFL thickness, mean VF MD, or visual field pattern SD between the MNG and control groups or between the NMG and MG groups.

### PPCMv analysis

Overall, there were significant differences among groups in mean PPCMv density across all ROIs (all P < 0.001). Univariable analysis showed that inner whole and outer whole annular PPCMv densities tended to decrease in the following order: control (mean [SD]: 0.15 [0.04] and 0.12 [0.04]), MNG (mean [SD]: 0.14 [0.02] and 0.12 [0.08]), NMG (mean [SD]: 0.09 [0.05] and 0.07 [0.04]), and MG (mean [SD]: 0.09 [0.04] and 0.07 [0.03]). Multivariable linear regression using a generalized estimating equation adjusted for age, sex, axial length, and VF MD showed significant differences in mean PPCMv density between glaucoma groups and the control group (mean difference [95% CI]: −0.06 [−0.08 to −0.04], P < 0.001 for inner whole annular PPCMv density in NMG vs control and −0.07 [−0.10 to −0.04], P < 0.001 for inner whole annular PPCMv density in MG vs control), consistent across all ROIs. In contrast, there were no significant differences in PPCMv density across all ROIs between the MNG and control groups (all P > 0.054). Finally, there was no interaction between glaucoma and myopia after adjustments for age, sex, axial length, and VF MD (P > 0.112) (Table 2).

**Table 2:** Differences in Inner Whole Annulus, Outer Whole Annulus, Inner Hemi-Superior, Outer Hemi-Superior, Inner Hemi-Inferior, and Outer Hemi-Inferior Annuli Between Experimental Groups and the Control Group.

	Inner Whole Annulus	P Value	Outer Whole Annulus	P Value	Inner Hemi-Superior	P Value	Outer Hemi-Superior	P Value	Inner Hemi-Inferior	P Value	Outer Hemi-Inferior	P Value
Control	Reference											
Myopia without glaucoma	−0.024 (−0.052 to 0.003)	0.086	−0.015 (−0.039 to 0.010)	0.244	−0.018 (−0.052 to 0.015)	0.292	−0.013 (−0.042 to 0.016)	0.389	−0.031 (−0.062 to 0.001)	0.054	−0.018 (−0.043 to 0.008)	0.182
Glaucoma without myopia	−0.064 (−0.085 to −0.044)	0.000	−0.052 (−0.070 to −0.034)	0.000	−0.074 (−0.10 to −0.05)	0.000	−0.057 (−0.079 to −0.035)	0.000	−0.056 (−0.079 to −0.033)	0.000	−0.048 (−0.066 to −0.029)	0.000
Myopia and glaucoma	−0.069 (−0.099 to −0.039)	0.000	−0.054 (−0.080 to −0.027)	0.000	−0.081 (−0.117 to −0.044)	0.000	−0.058 (−0.090 to −0.026)	0.000	−0.057 (−0.091 to −0.023)	0.001	−0.053 (−0.081 to −0.026)	0.000
Interaction between myopia and glaucoma	0.020 (−0.012 to 0.052)	0.226	0.012 (−0.016 to 0.041)	0.391	0.010 (−0.03 to 0.05)	0.585	0.010 (0.02 to 0.05)	0.501	0.03 (−0.01 to 0.07)	0.112	0.012 (−0.018 to 0.041)	0.438
Glaucoma without myopia	Reference											
Myopia without glaucoma	0.040 (0.012 to 0.068)	0.005	0.037 (0.013 tot 0.061)	0.003	0.056 (0.022 to 0.890)	0.001	0.044 (0.015 to 0.073)	0.003	0.025 (−0.006 to 0.056)	0.114	0.030 (0.005 to 0.055)	0.021
Myopia and glaucoma	−0.004 (−0.030 to 0.022)	0.738	−0.002 (−0.025 to 0.021)	0.857	−0.007 (−0.039 to 0.024)	0.658	−0.001 (−0.029 to 0.026)	0.936	−0.001 (−0.031 to 0.028)	0.924	−0.006 (−0.030 to 0.018)	0.635

Data are presented as means (95% confidence intervals).

Analyzed using a marginal model of generalized estimating equations adjusted for age, sex, axial length, and visual field mean deviation.

There were no factors significantly associated with inner and outer whole annuli PPCMv densities, according to mixed-effect multilevel regression analyses stratified by laterality (P > 0.096) (Table 3).

**Table 3:** Determinants of Parapapillary Choroidal Microvasculature in Glaucoma and Myopia.

	Glaucoma			Myopia		
	Mean Difference	P Value*	95% Confidence Interval	Mean Difference	P Value*	95% Confidence Interval
Inner Whole Annulus						
Age	0.000	0.485	−0.001 to 0.001	−0.002	0.114	−0.005 to 0.001
Axial length	0.003	0.232	−0002 to 0.009	0.005	0.502	−0.009 to 0.019
Sex	0.002	0.872	−0.018 to −0.021	−0.002	0.918	−0.042 to 0.038
VF MD	−0.001	0.096	−0.003 to 0.000	−0.001	0.676	−0.006 to 0.004
Outer Whole Annulus						
Age	0.000	0.300	−0.00 to 0.001	−0.002	0.154	−0.004 to 0.001
Axial length	0.000	0.990	−0.005 to 0.005	0.004	0.525	−0.009 to 0.018
Sex	−0.003	0.703	−0.021 to 0.014	−0.001	0.947	−0.039 to 0.036
VF MD	0.000	0.546	−0.000 to −0.001	0.000	0.854	−0.004 to 0.005

*P Value indicates.

VF, visual field; MD, mean deviation

## Discussion

Because OCTA-based evaluations of glaucoma are not affected by low reflectance of the RNFL or structural deformation of the optic disc, this study used OCTA to assess choroidal blood flow in myopic eyes with or without glaucoma. Automated customized software revealed a progressive decline in PPCMv density across groups in the following order: control, MNG, NMG, and MG. The mean difference in PPCMv density between glaucoma groups and the control group was statistically significant and consistent across all ROIs. No interaction was detected between glaucoma and myopia.

The relationship between myopia and decreased vessel density is well-established. Myopic eyes exhibit altered retinal circulation with narrower vessels, altered bifurcation, and reductions in central retinal artery diameter and flow [14]. We previously identified a general pattern of decreases in parapapillary vessel density among groups, in the following order: control, myopia only, glaucoma only, and myopia combined with glaucoma [16]. In addition to the microvascular attenuation evident with myopia or OAG alone, a larger degree of attenuation is observed in patients with both conditions. Our previous results also indicated that, compared with glaucoma, myopia has a weaker effect on parapapillary vessel density [16].

In the present study, we observed a similar tendency for decreases in PPCMv density among the groups. Choroidal microvascular dropout, observed in 97.8% of primary OAG eyes with high myopia, has been associated with the presence of central scotoma [22,23]. Parapapillary choroidal microvascular dropouts are prevalent in highly myopic primary OAG eyes; they display topographic correlations with the locations of glaucomatous VF defects despite potential inaccuracies regarding RNFL thickness due to segmentation errors [22]. Lin et al. recently reported that primary OAG eyes showed reduced superficial choroidal capillary density, whereas highly myopic eyes showed reduced deep choroidal capillary density in the macular region [24]. However, there have been conflicting results concerning choroidal blood flow measurements by color Doppler imaging in myopia patients without and with glaucoma [25]. Posterior ciliary artery velocities demonstrated similar decreases, such that there were no significant differences between myopic eyes with and without glaucoma. These conflicting results may be related to variations in blood flow measurement techniques and specific vessels analyzed. Additionally, OCTA devices are designed to measure vessel perfusion, rather than the actual flow rates obtained by color Doppler imaging. Hu et al. reported that choroidal microvascular density was lower in patients with beta-zone parapapillary atrophy than in patients with gamma-zone parapapillary atrophy (P < 0.01). Choroidal microvascular densities in beta and gamma zones were negatively correlated with the widths of those respective zones. In the present study, there were no factors significantly associated with inner and outer whole annuli PPCMv densities.

Comparisons of the vascular characteristics of glaucoma patients with and without myopia revealed that eyes in the MG group displayed more pronounced vascular alterations compared with eyes in the NMG group. These alterations included greater reductions in choroidal blood flow and velocity, lower capillary densities in the macular and parapapillary regions, and impaired vasoreactivity in the parapapillary area. Thus, we hypothesize that the association between myopia and glaucoma is partly attributed to vascular factors, particularly microvascular alterations; these factors may precede the onset of evident glaucomatous damage.

It has been reported that the development and progression of glaucoma are more aggressive in highly myopic eyes than in minimally myopic or non-myopic eyes. Furthermore, the severity of decreases in retinal microcirculation has been correlated with axial length and thinning of the ocular wall in glaucoma patients; longer axial lengths and thinner ocular walls were associated with greater reduction [26].

## Limitations

This study had some limitations. First, the presence of choroidal atrophy, Bruch’s membrane rupture, and posterior staphyloma in myopic axial elongation are obstacles of detection with automated segmentation provided by OCTA. To exclude the effects of such artifacts, we excluded eyes that exhibited segmentation errors within the ROIs. The presence of myopic changes in the posterior pole could also affect choroidal vascularity; this effect should be considered in future studies [27].

Second, the small number of patients may affect the generalizability of the findings and restrict their application at the population level. Considering the complex dynamics of myopia and glaucoma, a larger and more diverse study population would help to ensure accurate representation of both conditions.

Finally, the cross-sectional design of this retrospective study hindered establishment of causality between the two conditions. The longitudinal observations of parapapillary microvasculature and microstructure may help to reveal relationships between axial elongation in myopia and OAG, with potential implications for clinical monitoring and management.

In summary, this study demonstrates a trend of progressive loss of PPCMv densities from control to MNG to NMG, to MG. Even though, this did not reach statistically significant level due to limit small sample size. In addition, we observed a similar tendency for decreases in RPC densities. The presence of myopia in combination with glaucoma could lead to greater damage than the result of elevated IOP in glaucoma alone. It remains unclear whether the reduced PPCMv density in myopia is a result of axial elongation or an aspect of myopia development; this issue requires further investigation.
