# Correction: Association of myopia and parapapillary choroidal microvascular density in primary open-angle glaucoma

**DOI:** 10.1371/journal.pone.0343933

**Published:** 2026-02-25

**Authors:** The PLOS One Staff

The corresponding author’s email address is incorrect. Wasu Supakontanasan’s email address is: wasu.supakontanasan@gmail.com.

In the Author Contributions, Hajar Danesh should be listed as one of the persons who contributed to Software and Writing – review & editing.

The publisher apologizes for the errors.
